# Supplementary material for: Functional Proteomics Characterization of the Role of SPRYD7 in Colorectal Cancer Progression and Metastasis
Source: Cells. 2023 Oct 31;12(21):2548. doi: 10.3390/cells12212548 (PMC10648221; doi:10.3390/cells12212548)
Supplement: Supplementary file 1 [file cells-12-02548-s001.zip › Revised Supplementary Figure 3.pptx]

## Slide 1
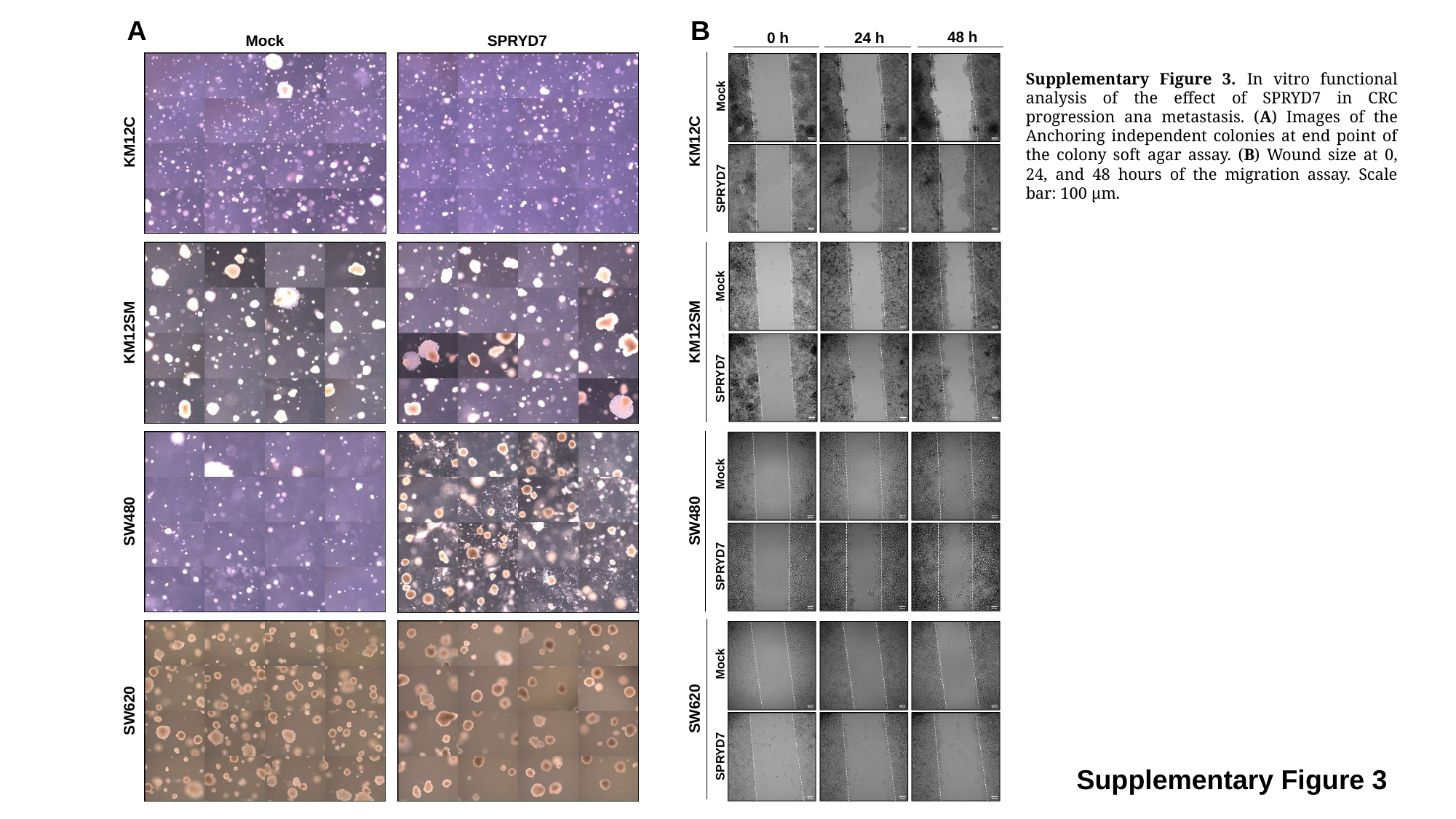

A
Mock
SPRYD7
KM12C
KM12SM
SW480
SW620
B
48 h
0 h
24 h
Mock
KM12C
SPRYD7
Mock
KM12SM
SPRYD7
Mock
SW480
SPRYD7
Mock
SW620
SPRYD7
Supplementary Figure 3. In vitro functional analysis of the effect of SPRYD7 in CRC progression ana metastasis. (A) Images of the Anchoring independent colonies at end point of the colony soft agar assay. (B) Wound size at 0, 24, and 48 hours of the migration assay. Scale bar: 100 µm.
Supplementary Figure 3
