# Supplementary material for: Functional Proteomics Characterization of the Role of SPRYD7 in Colorectal Cancer Progression and Metastasis
Source: Cells. 2023 Oct 31;12(21):2548. doi: 10.3390/cells12212548 (PMC10648221; doi:10.3390/cells12212548)
Supplement: Supplementary file 1 [file cells-12-02548-s001.zip › Supplementary Figure 1.pptx]

## Slide 1
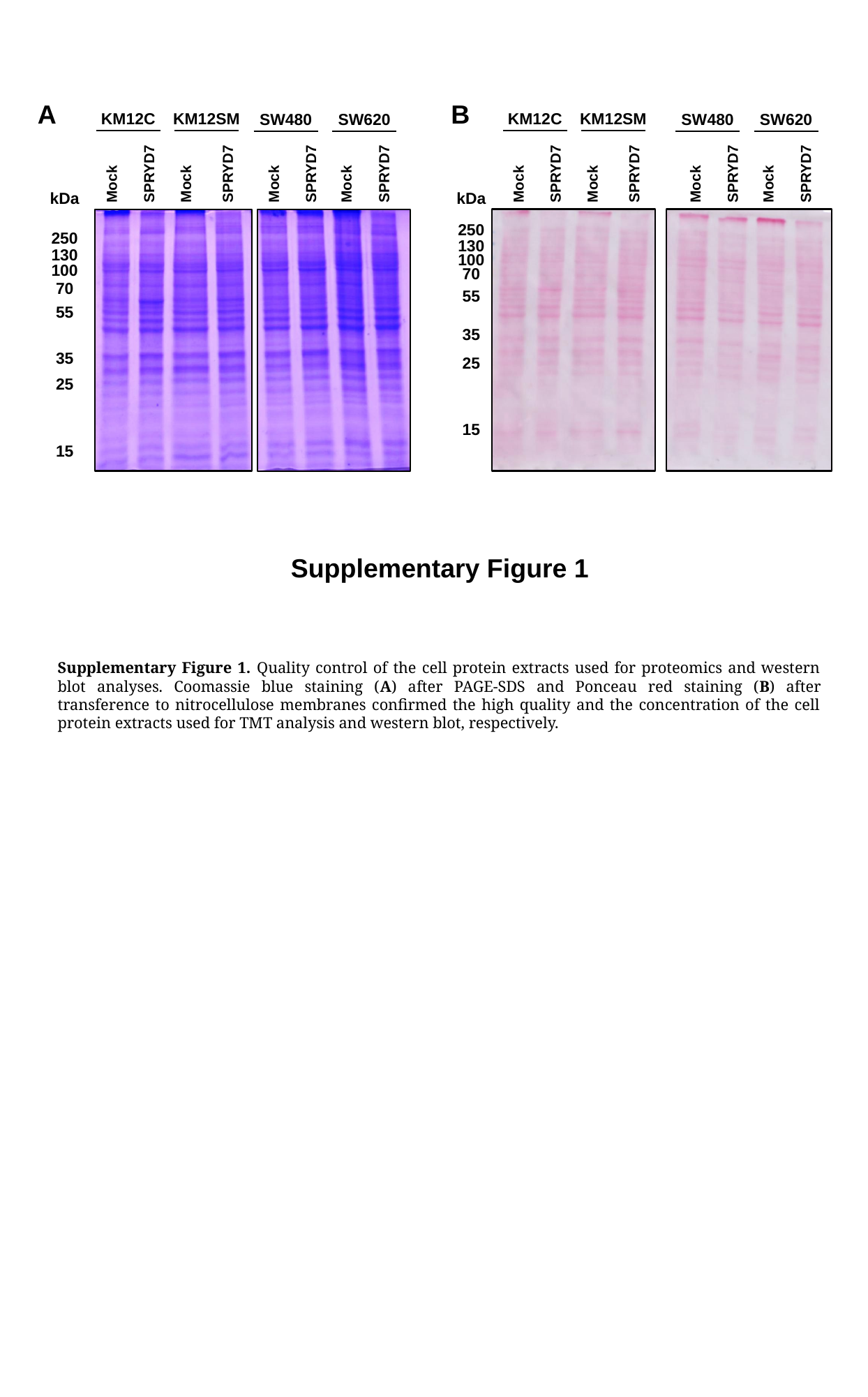

A
B
KM12C
KM12SM
KM12C
KM12SM
SW480
SW620
SW480
SW620
Mock
SPRYD7
Mock
SPRYD7
Mock
SPRYD7
Mock
SPRYD7
Mock
SPRYD7
Mock
SPRYD7
Mock
SPRYD7
Mock
SPRYD7
kDa
kDa
250
250
130
130
100
100
70
70
55
55
35
35
25
25
15
15
Supplementary Figure 1
Supplementary Figure 1. Quality control of the cell protein extracts used for proteomics and western blot analyses. Coomassie blue staining (A) after PAGE-SDS and Ponceau red staining (B) after transference to nitrocellulose membranes confirmed the high quality and the concentration of the cell protein extracts used for TMT analysis and western blot, respectively.
