# Supplementary material for: Functional Proteomics Characterization of the Role of SPRYD7 in Colorectal Cancer Progression and Metastasis
Source: Cells. 2023 Oct 31;12(21):2548. doi: 10.3390/cells12212548 (PMC10648221; doi:10.3390/cells12212548)
Supplement: Supplementary file 1 [file cells-12-02548-s001.zip › Supplementary Figure 2.pptx]

## Slide 1
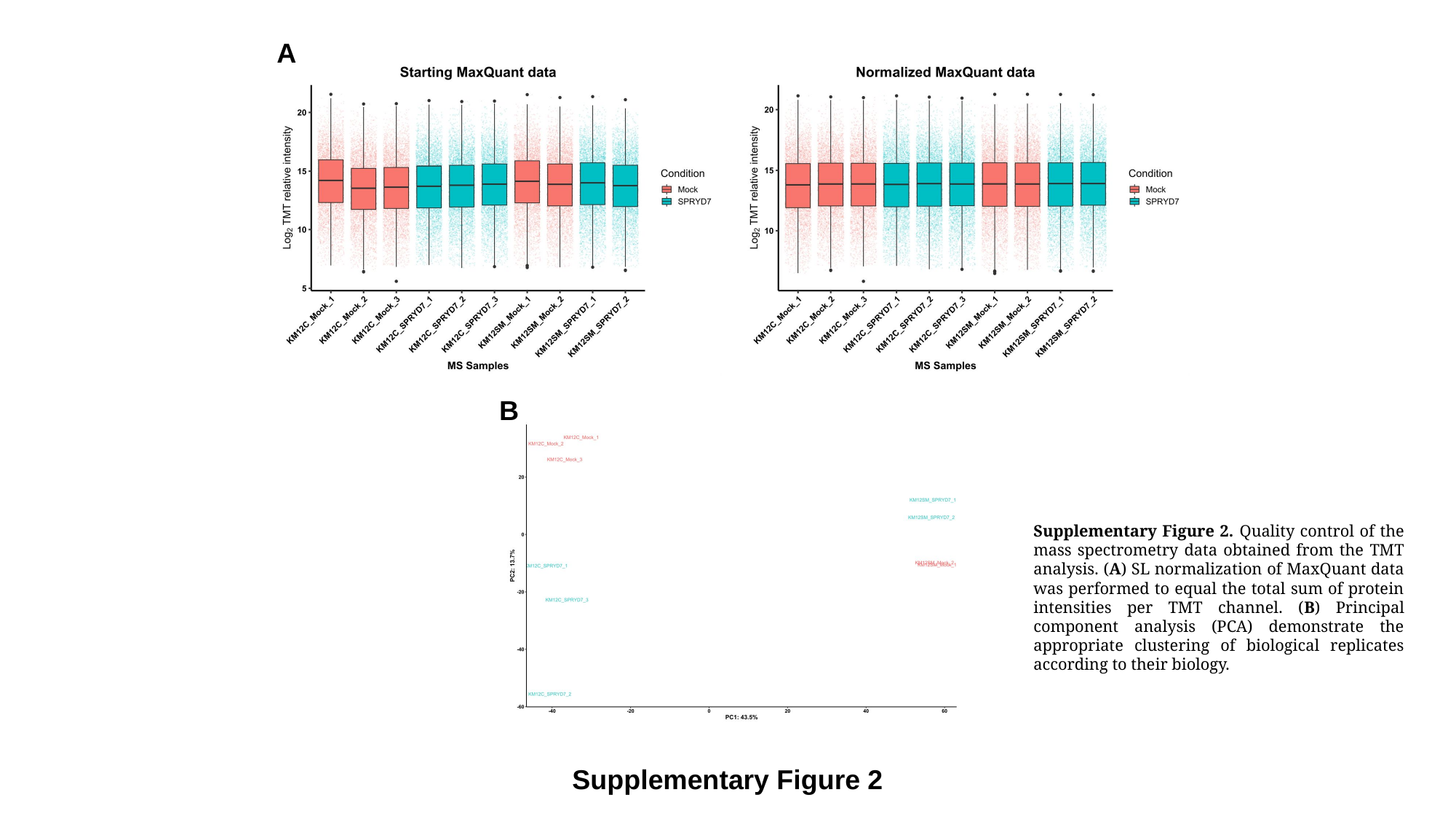

A
B
Supplementary Figure 2. Quality control of the mass spectrometry data obtained from the TMT analysis. (A) SL normalization of MaxQuant data was performed to equal the total sum of protein intensities per TMT channel. (B) Principal component analysis (PCA) demonstrate the appropriate clustering of biological replicates according to their biology.
Supplementary Figure 2
