# Supplementary material for: Functional Proteomics Characterization of the Role of SPRYD7 in Colorectal Cancer Progression and Metastasis
Source: Cells. 2023 Oct 31;12(21):2548. doi: 10.3390/cells12212548 (PMC10648221; doi:10.3390/cells12212548)
Supplement: Supplementary file 1 [file cells-12-02548-s001.zip › Revised Supplementary Figure 4.pptx]

## Slide 1
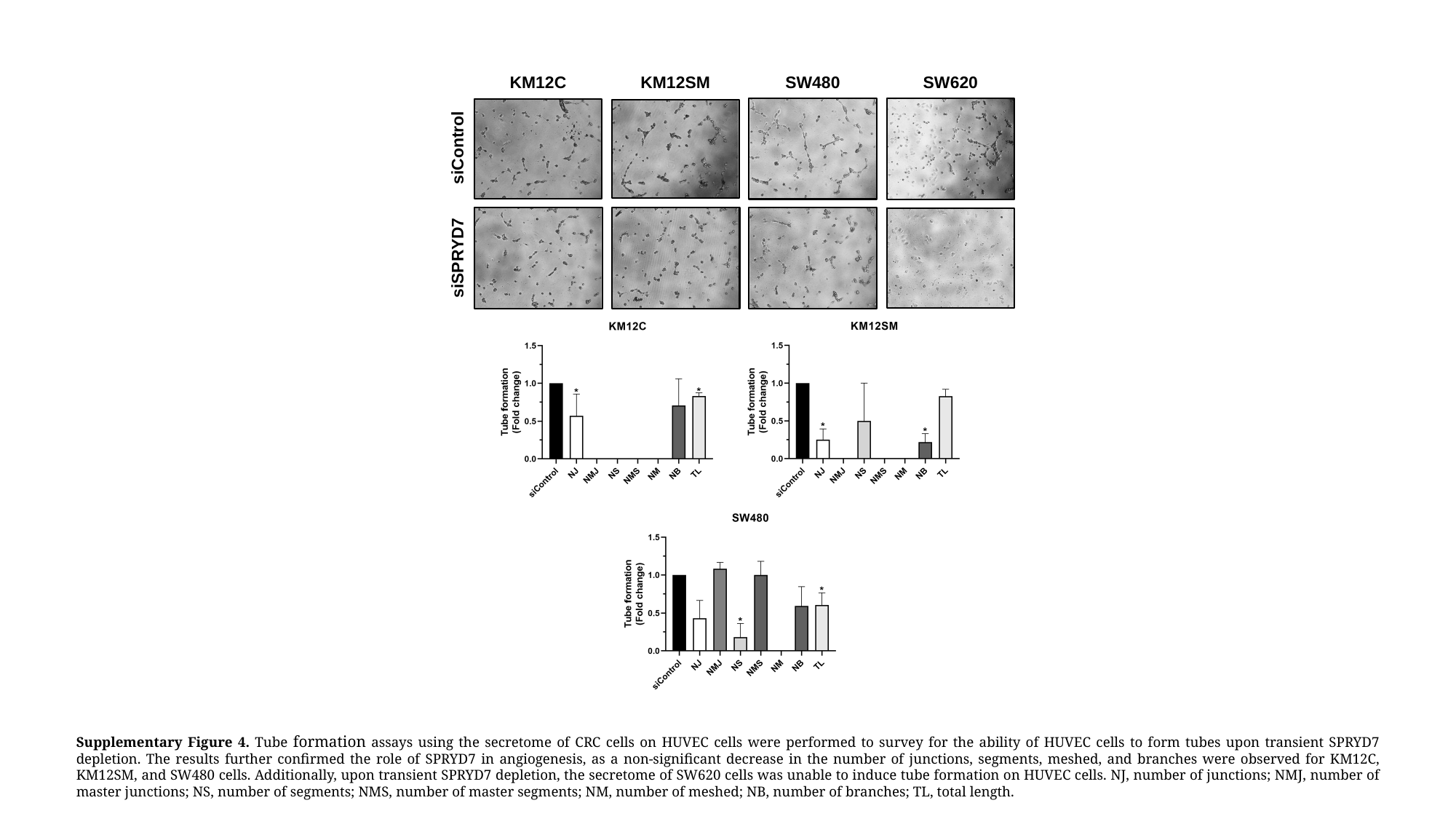

SW620
KM12C
KM12SM
SW480
siControl
siSPRYD7
Supplementary Figure 4. Tube formation assays using the secretome of CRC cells on HUVEC cells were performed to survey for the ability of HUVEC cells to form tubes upon transient SPRYD7 depletion. The results further confirmed the role of SPRYD7 in angiogenesis, as a non-significant decrease in the number of junctions, segments, meshed, and branches were observed for KM12C, KM12SM, and SW480 cells. Additionally, upon transient SPRYD7 depletion, the secretome of SW620 cells was unable to induce tube formation on HUVEC cells. NJ, number of junctions; NMJ, number of master junctions; NS, number of segments; NMS, number of master segments; NM, number of meshed; NB, number of branches; TL, total length.
