# Supplementary material for: Functional Proteomics Characterization of the Role of SPRYD7 in Colorectal Cancer Progression and Metastasis
Source: Cells. 2023 Oct 31;12(21):2548. doi: 10.3390/cells12212548 (PMC10648221; doi:10.3390/cells12212548)
Supplement: Supplementary file 1 [file cells-12-02548-s001.zip › Revised Supplementary Figure 5.pptx]

## Slide 1
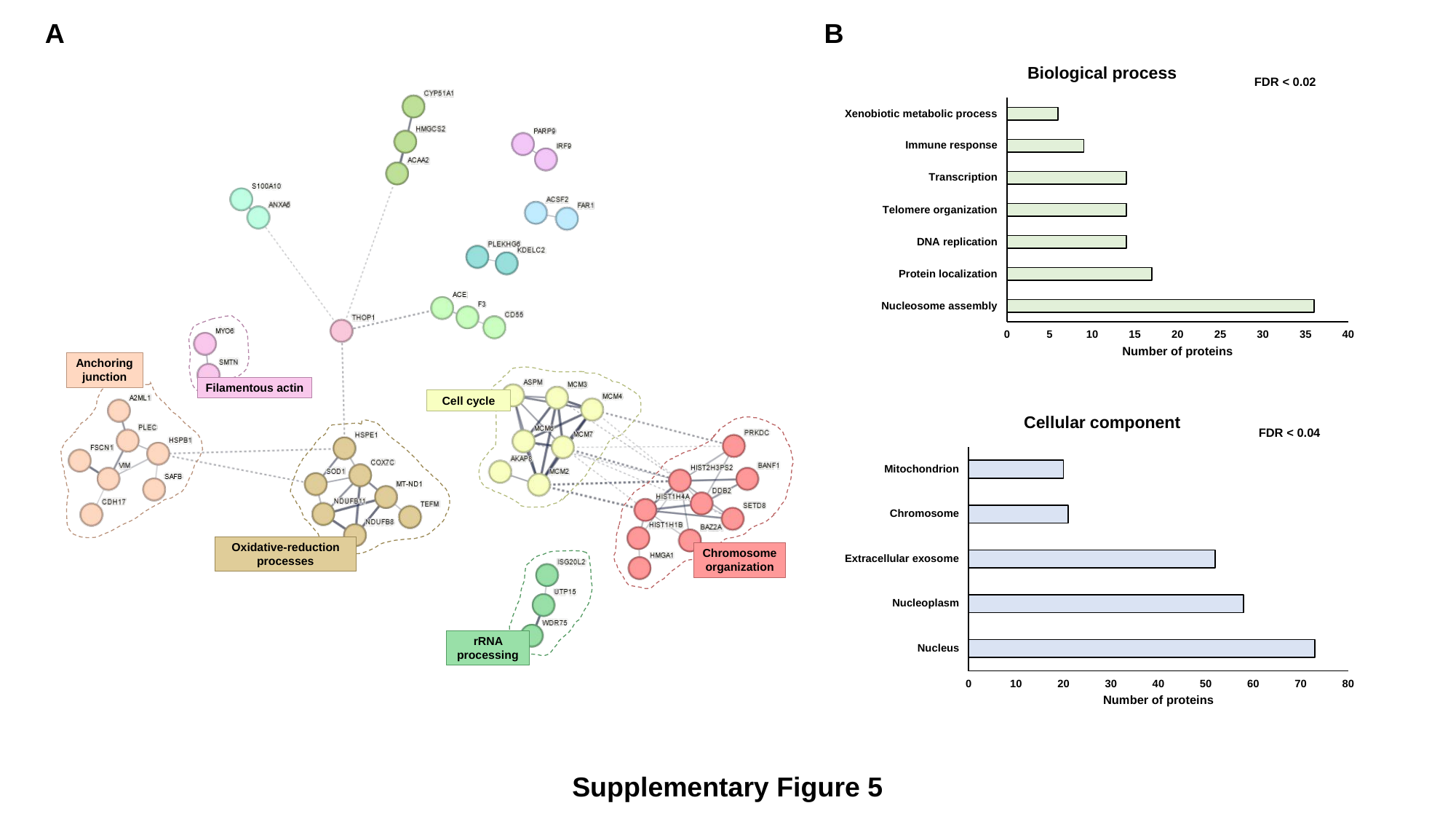

A
B
Supplementary Figure 5
Supplementary Figure 5. Bioinformatics analysis of proteins dysregulated by SPRYD7 overexpression. (A) STRING analysis revealed more than 16 clusters of direct and indirect interaction among dysregulated proteins related to CRC, such as metabolic processes, immune response, cell junction, migration, or regulation of cytoskeleton. (B) Protein enrichment with DAVID database confirmed the association of dysregulated proteins with processes involved in CRC, such as transcription, DNA replication, immune response, or protein localization, and revealed the cytoplasm, nucleus, and exosomes as the main cellular localization of dysregulated proteins.
