# Supplementary material for: Functional Proteomics Characterization of the Role of SPRYD7 in Colorectal Cancer Progression and Metastasis
Source: Cells. 2023 Oct 31;12(21):2548. doi: 10.3390/cells12212548 (PMC10648221; doi:10.3390/cells12212548)
Supplement: Supplementary file 1 [file cells-12-02548-s001.zip › Revised Supplementary Figure 6.pptx]

## Slide 1
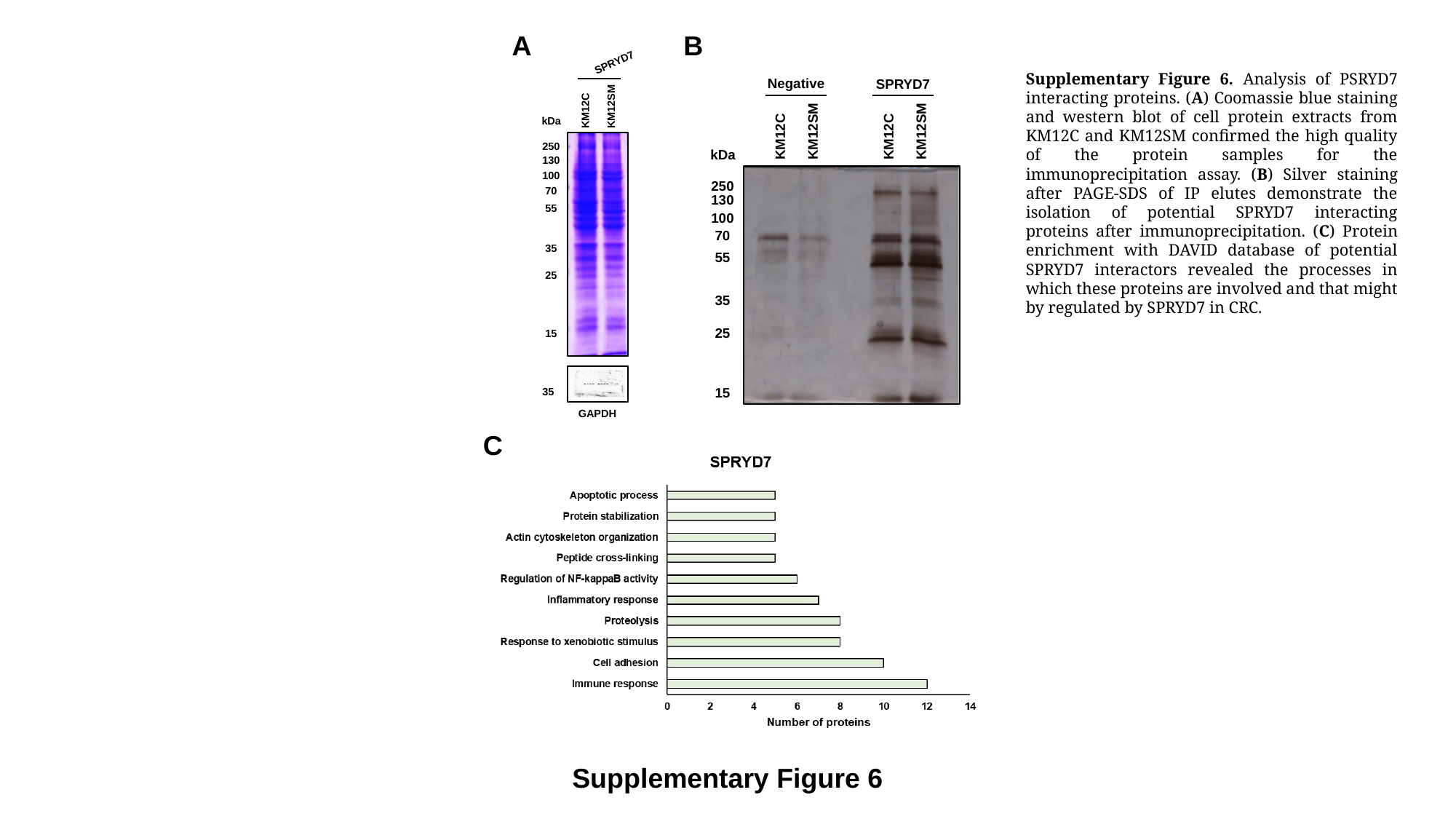

A
B
SPRYD7
KM12SM
KM12C
kDa
250
130
100
70
55
35
25
15
35
GAPDH
Negative
SPRYD7
KM12SM
KM12SM
KM12C
KM12C
kDa
250
130
100
70
55
35
25
15
Supplementary Figure 6. Analysis of PSRYD7 interacting proteins. (A) Coomassie blue staining and western blot of cell protein extracts from KM12C and KM12SM confirmed the high quality of the protein samples for the immunoprecipitation assay. (B) Silver staining after PAGE-SDS of IP elutes demonstrate the isolation of potential SPRYD7 interacting proteins after immunoprecipitation. (C) Protein enrichment with DAVID database of potential SPRYD7 interactors revealed the processes in which these proteins are involved and that might by regulated by SPRYD7 in CRC.
C
Supplementary Figure 6
